# Health Disparities in Genomics and Genetics

**DOI:** 10.1155/2014/324327

**Published:** 2014-04-22

**Authors:** Ida J. Spruill, Jacquelyn Taylor, Irma B. Ancheta, Adebowale A. Adeyemo, Yolanda Powell-Young, Willa Doswell

**Affiliations:** ^1^Medical University of South Carolina, Charleston, SC 29407, USA; ^2^Yale School of Nursing, Orange, CT 06477, USA; ^3^School of Nursing, Brooks College of Health, University of North Florida, Jacksonville, FL 32224, USA; ^4^National Institute of Health, National Human Genome Research Institute, and Center for Research on Genomics and Global Health, Bethesda, MD 20892-5635, USA; ^5^Center for Minority Health and Health Disparities Research, Dillard University and LSU Health Sciences Center Collaborative, New Orleans, LA 70122, USA; ^6^Department of Health Promotion, School of Nursing, University of Pittsburgh, Pittsburg, PA 15261, USA

Disparities or inequities in health refer to sociodemographic group differences in the distribution of disease, health outcomes, or access to health care [1]. In order to eliminate health disparities, more efforts are needed to address social issues directly contributing to the healthy inequities observed across racial and ethnic groups. With broad support from many federal agencies, alleviating health disparities in the United States remains a goal of Healthy People 2010 [2]. However, genetic research also has a significant role to play in alleviating and understanding disparities. With tremendous advances in technology and increased investigation into human genetic variations, genomics is poised to play a valuable role in bolstering efforts to find new treatments and preventions for chronic conditions that disparately affect certain ethnic groups. The recent statement regarding the future of genomics from the National Human Genome Research Institute (NHGRI) [3] indicated that the need to develop genome-based tools to address health disparities remains a “grand challenge”. The statement acknowledges that social and economic factors contribute significantly to disparities but nevertheless assert the need for extensive research to better understand the contribution of genetics.

In this special issue, we present five promising studies conducted by nurse scientists focused on understanding the genetic underpinnings of diseases such as epigenetic markers of renal function in African Americans and the association between KIF6 single nucleotides in Filipino women. Other studies reported on participation of African Americans into research, attitudes toward genetic testing for hypertension, and, lastly, the role of epigenetics in health disparity among Native Americans. All have characterized the importance of their work in its capacity to advance our understanding of health disparities research.

The resounding success of the human genome project (HGP), international hap map project, and the 1000 genomes project has brought clinical translation closer than ever. Although some prevention programs now exist to reduce disparities by targeting specific genetic disorders, publications by ethnic minorities of nurses remains underrepresented in genetic research. In seeking articles for this special edition, the editors actively engage authors to submit publications addressing health disparities in genomics and genetics. Although there is an interest in the topic and it is evident globally with Journal of Nursing Scholarship, it is still an underresearched area. Nevertheless, it is a topic that is acknowledged to have great importance to understanding the underpinnings of disparity in genetics and genomics.

More specifically, this special edition is dedicated to genetic/genomic research conducted by nurse scientist like V. Ancheta et al. entitled “*the association between KIF6 single nucleotide polymorphism rs20455 and serum lipids in Filipino-American women.*” Their results showed that the majority of Filipino women are either heterozygous or homozygous carriers of the risk allele of the rs20455 SNP in the KIF6 gene. More importantly, even with borderline LDL-C levels, many FA would benefit from statin treatment, and many participants did not exhibit guideline recommended LDL-C levels including those on statin drugs.

On the other hand, work by S. M. Bomotti et al. entitled “*epigenetic markers of renal function in African Americans*” focused on chronic kidney disease (CKD) in African Americans. The authors sought to understand how DNA methylation plays a role in CKD. To better understand the role of these methylation markers, they measured 26,428 DNA methylation sites in 972 African Americans from the genetic epidemiology network of arteriopathy (GENOA) study to evaluate (1) whether epigenetic markers (EMs) are associated with estimated glomerular filtration rate (eGFR), (2) whether the significantly associated markers are also associated with traditional risk factors and/or novel biomarkers for eGFR, and (3) how much additional variation in eGFR is explained by epigenetic markers. The authors concluded that at least six EM were identified to predict eGFR.

Most importantly, innovative work by T. M. Brockie et al. entitled “*a framework to examine the role of epigenetics in health disparities among Native Americans*” offers an interesting insight to stress among Native Americans. They reported that Native Americans disproportionately experience adverse childhood experiences (ACEs) as well as health disparities, including high rates of posttraumatic stress, depression, and substance abuse, and that many ACEs have been linked to methylation changes in genes that regulate the stress response, thus suggesting that these molecular changes may underlie the risk for psychiatric disorders. The authors reviewed published studies to provide evidence that ACEs-related methylation changes contribute to health disparities and that this framework may be adapted to understand how ACEs may result in health disparities in other racial/ethnic groups.

S. M. Underwood et al. were interested in factors that may prevent involvement of African Americans' participation in genetic research. Their study entitled“*enhancing the participation of African Americans in health-related genetic research: findings of a collaborative academic and community-based research stud*y” sought to identify factors inhibiting the participation of African Americans in health-related research. They employed an exploratory study of factors presumed to be associated with participation in health-related research, among a nonprobability sample of African Americans from a large urban community in the Midwest. The results revealed that knowledge, beliefs, and perceptions about genetics and the involvement of providers were associated with willingness to engage in health-related genetic research. The most interesting, however, was that 88.7% of the participants reported that “they had never been asked” to participate in research.

Similarly, J. Y. Taylor et al. were concerned about the high prevalence of hypertension among African American women. Their study entitled “*attitudes toward genetic testing for hypertension among African American women and girls*” sought to understand perceived barriers and benefits for genetic testing of multigenerational triads. They employed the health belief model as a guide to examine attitudes toward perceived barriers and benefits of genetic testing and to determine whether they differed by generation, age, education, or income level. Results indicated that increased age and education were associated with significant differences in attitudes regarding benefits. They highlighted the need for increased outreach to younger generations regarding benefits of genetic services.

These articles illustrate and summarize many of the ways in which nurse scientists are involved in disparities around genetics and genomic research. Understandably, some medical research adopts well-established perspective that racial discrimination and poverty are the major contributors to unequal health status. Therefore programs advocating social justices must be supported.

The editors of this special topic issue hope that these studies will stimulate additional discussion around genetic variation between and among population groups, the role of epigenetics in understanding genes expression of chronic diseases, and the inequalities observed among “racialized groups.”

The overall intention of the editors is that other nurses will become proactive in understanding the relationship between gene expression, environment, and illness. It is critical that research on the genetics of human health and disease continue and bring to clinical fruition the vast storehouse of basic and observational research that the human genome project has produced.

*Ida J. Spruill* *Ida J. Spruill* *Jacquelyn Y. Taylor* *Jacquelyn Y. Taylor* *Irma B. Ancheta* *Irma B. Ancheta* *Adebowale A. Adeyemo* *Adebowale A. Adeyemo* *Yolanda Powell-Young* *Yolanda Powell-Young* *Willa Doswell* *Willa Doswell*
